# The associations of gut microbiota, endocrine system and bone metabolism

**DOI:** 10.3389/fmicb.2023.1124945

**Published:** 2023-04-06

**Authors:** Ye Tu, Xinyi Kuang, Ling Zhang, Xin Xu

**Affiliations:** ^1^State Key Laboratory of Oral Diseases, National Clinical Research Center for Oral Diseases, West China Hospital of Stomatology, Sichuan University, Chengdu, China; ^2^Department of Cariology and Endodontics, West China Hospital of Stomatology, Sichuan University, Chengdu, China

**Keywords:** gut microbiota, bone metabolism, gut-bone axis, endocrine system, hormone

## Abstract

Gut microbiota is of great importance in human health, and its roles in the maintenance of skeletal homeostasis have long been recognized as the “gut-bone axis.” Recent evidence has indicated intercorrelations between gut microbiota, endocrine system and bone metabolism. This review article discussed the complex interactions between gut microbiota and bone metabolism-related hormones, including sex steroids, insulin-like growth factors, 5-hydroxytryptamine, parathyroid hormone, glucagon-like peptides, peptide YY, etc. Although the underlying mechanisms still need further investigation, the regulatory effect of gut microbiota on bone health *via* interplaying with endocrine system may provide a new paradigm for the better management of musculoskeletal disorders.

## 1. Introduction

Known as the “second gene pool” of the human body, gut microbiota plays important roles in human health, and the dysbiosis of gut microbiota is closely involved in a variety of diseases, including gastrointestinal diseases (Hold, 2016; Ni et al., 2017), cardiovascular disease (Tang et al., 2017; Wang and Zhao, 2018), metabolic disorder (Maruvada et al., 2017; Makki et al., 2018), and psychiatric disorder (Mangiola et al., 2016; Fattorusso et al., 2019).

Numerous studies have demonstrated the critical roles of gut microbiota in regulating bone homeostasis. By using high-throughput sequencing, the alteration of gut microbiota has been detected in individuals with osteopenia or osteoporosis, characterized with an increased abundance of lipopolysaccharides (LPS)-producing genera (Das et al., 2019; Li et al., 2019; Wei et al., 2021). Germ-free (GF) animals or animals treated with antibiotics have been widely used to investigate the potential roles of microbiota in bone metabolism, revealing the regulatory effect of commensal microbiota on bone mineral density (BMD) and bone property (Sjögren et al., 2012; Guss et al., 2017; Castaneda et al., 2020). In addition, gut microbiota can regulate skeletal system by affecting nutrition absorption, gut barrier functionality and immune response (Lafage Proust, 2017; Li et al., 2021; Tu et al., 2021). Recently, although direct causal effect has not been demonstrated, the regulatory effect of gut microbiota on skeletal homeostasis dependent on the endocrine system, known as “enteroendocrine-osseous axis,” has drawn increasing attentions. In this review, we discussed the evidence of hormone-bone interactions and potential mechanisms of how gut microbiota regulates the enteroendocrine-osseous axis, based on current evidence in this field.

## 2. Clinical evidences of the association between endocrine system and bone health

Since a large part of patients with endocrine diseases are accompanied by skeletal disorders, the interaction between endocrine activity and bone metabolism has come into notice. Hepatic osteodystrophy refers to bone diseases in patients with chronic liver disease, and approximately 30% of patients with chronic liver disease suffer from osteoporosis (Lehto-Axtelius et al., 2002; Collier, 2007). Patients who received liver transplants may suffer from accelerated bone loss and elevated fracture rate in the short term after the surgery (European Association for the Study of the Liver, 2016; Kovvuru et al., 2020). Hepatocellular dysfunction leads to the synthesis defect of growth hormone (GH) and insulin-like growth factor 1 (IGF-1), and the endocrine abnormality is perceived as the causation of bone metabolic complications (Baruch et al., 1998; Held et al., 2005; Assy et al., 2008). Chronic kidney disease (CKD) is associated with the development of the mineral and bone disorder (MBD), osteoporosis, and fragility fracture, characterized with abnormal serum levels of calcium, phosphorus, parathyroid hormone (PTH), and vitamin D (Hsu et al., 2020; Pazianas and Miller, 2021). As for primary hyperparathyroidism (PHPT), excessive endogenous PTH promotes bone turnover and results in cortical and trabecular bone loss (Mosekilde, 2008; Silva et al., 2011). Interestingly, being the complication of PHPT and CKD, the aggravated bone turnover generally caused by enhanced PTH level does not always occur as the disease advances (Pierreux and Bravenboer, 2018; Palermo et al., 2020). Additionally, the absence of bone destruction after continuous PTH (cPTH) administration was observed in GF mice (Yu et al., 2020). These findings indicate that gut microbiota might serve as a potential mediator in the enteroendocrine-osseous axis.

## 3. Gut microbiota and enteroendocrine system: Joint effects on bone metabolism

### 3.1. Sex steroids

Osteoporosis (OP) is the most prevalent skeletal disorder characterized by decreased BMD and bone microarchitecture destruction, which mainly occurs in elderly people, typically postmenopausal women (Lane et al., 2000; Johnston and Dagar, 2020). Strong benefits of sex steroids in bone metabolism have long been recognized. Estrogen is of great importance in bone homeostasis. In addition to directly regulating the development of osteoblasts and osteoclasts *via* Fas/Fas ligand pathway (Nakamura et al., 2007; Krum et al., 2008), estrogen prevents bone resorption by maintaining systemic and bone marrow T cells homeostasis (Cenci et al., 2003; Jia et al., 2021). Recent studies have revealed the correlations between gut microbiota and postmenopausal osteoporosis (PMO), as gut microbiota dysbiosis was observed under the condition of sex steroid deficiency, whereas the estrogen deficiency-induced bone destruction was not observed in GF mice (Li et al., 2016; He et al., 2020; Wang et al., 2021; Yang et al., 2022). Intricate regulatory effects of gut microbiota in PMO have been demonstrated. On one hand, elevated LPS level caused by gut microbial dysbiosis can stimulate the secretion of inflammatory cytokines, including IL-1β, IL-6, TNF-α, thus promoting bone resorption (Islam et al., 2007; Bandow et al., 2010; Xie et al., 2014; Hirano et al., 2017). On the other hand, short chain fatty acids (SCFAs) derived from gut microbiota exert bone-protective effects by inducing the generation of regulatory T cells (Tregs) (Furusawa et al., 2013; Dalile et al., 2019) and suppressing Th17 cells (Asarat et al., 2016; Luu et al., 2019). Being the most common modulator of gut microbiota, probiotics have been demonstrated to ameliorate estrogen deficiency-induced bone loss in rodents *via* promoting SCFAs production, restoring the gut permeability, and reducing inflammatory response (Li et al., 2016; Jia et al., 2021). The favorable effects of probiotics on bone health have also demonstrated in clinical trials (Narva et al., 2004; Jafarnejad et al., 2017; Lambert et al., 2017). In addition, it has been shown that administration of brain progesterone can effectively relive the bone loss while maintaining the diversity of the gut microbiomes in estrogen-deficient rats (Park et al., 2018).

Low testosterone level in hypogonadal men is also associated with decreased BMD, and testosterone treatment can effectively improve the bone structure (Kenny et al., 2001; Colleluori et al., 2021). It has been demonstrated that testosterone can stimulate the proliferation of osteoblast precursors, and as well suppress the osteoblasts apoptosis (Damien et al., 2000; Wiren et al., 2006). Approaches that influence gut environment, including probiotics administration and fecal microbiota transplanting, can regulate testosterone level (Flores et al., 2012; Markle et al., 2013), and concurrently alter bone structure (Ai et al., 2021; Han et al., 2022). However, further investigations are still needed to reveal the underlying mechanisms.

### 3.2. GH/ IGF-1 axis

The longitudinal bone growth resulting from endochondral ossification is regulated by the GH/insulin-like growth factor (IGF) axis (Wong et al., 2016). IGFs are required for cell survival, proliferation and differentiation, and IGF-1 has long been recognized as a pivotal candidate in osteoblastogenesis (Stewart and Rotwein, 1996; Van Wyk and Smith, 1999). The bone quality is strongly linked to the serum IGF-1 level (van Coeverden et al., 2002; Rosen et al., 2004; Fazeli et al., 2020; Delagrange et al., 2021), and the circulating IGF-1 is mainly synthesized by liver under the regulation of GH (Stewart and Rotwein, 1996; Van Wyk and Smith, 1999). Besides, local production of IGF-1 from osteoblasts, osteoclasts and osteocytes are capable of promoting osteoblastic activity and bone formation, demonstrating the great importance of paracrine/autocrine functions of IGF-1 in bone metabolism (Zhao et al., 2000; Hamrick, 2012; Wang et al., 2013; Kirk et al., 2020). IGF-1 is also required for osteoclastogenesis. Mature osteoclasts express IGF-1 receptor, and IGF-1 deprivation leads to reduction in osteoclast number, size and function, as well as decreased expression of RANKL and RANK (Hou et al., 1997; Wang et al., 2006).

The roles of microbiota in anabolic effects of IGF-1 have been observed in a wide variety of species. In *Drosophila*, commensal bacteria are responsible for the production of *Drosophila* insulin-like peptides (dILPs), analogs of mammalian insulin and IGFs, and dILPs control the duration of the larval period as well as the larval growth rate (Hietakangas and Cohen, 2009; Kannan and Fridell, 2013). Axenic fly larvae suffer from defected development, while colonization of *Acetobacter pomorum* effectively restore the body development and promoted *DILP2* expression in larvae (Shin et al., 2011). By optimizing diet-derived branched-chain amino acids (BCAAs) level, the colonization of *Lactobacillus plantarum* stimulates the TOR kinase activity, leading to increased dILPs production in brain with elevated growth rate (Storelli et al., 2011). In the rodent model, a significant reduction of circulating GH and IGF-1 levels has been observed in both GF and axenic mice (Schwarzer et al., 2016; Yan et al., 2016; Weger et al., 2019), while the colonization of GF mice with gut microbiota from conventional raised mice or *Lactobacillus plantarum* strains can effectively restore the serum level of IGF-1 (Schwarzer et al., 2016; Yan et al., 2016). Early intestinal intervention with fecal microbiota transplantation promotes growth and elevates serum GH and IGF-1 levels in suckling piglets (Cheng et al., 2019). In addition, the presence of gut microbiota also increases the bone marrow IGF-1 level, indicating the enhanced autocrine and paracrine activity of IGF-1 in bone remodeling (Yan et al., 2016). Of note, the impacts of gut microbiota on the GH/IGF-1 axis depend on the duration of colonization and animal age. A reduced trabecular bone quantity and serum IGF-1 level has been observed in SPF mice as compared to that in GF mice (Novince et al., 2017), and accelerated bone resorption has also been detected after short-term colonization of gut microbiota to GF mice (Yan et al., 2016).

### 3.3. Parathyroid hormone

Produced by parathyroid glands, parathyroid hormone (PTH) is an important regulator of calcium metabolism, presenting as a double-edged sword to skeletal homeostasis. The administration of continuous PTH (cPTH) leads to increased level of receptor activator of NF-κB ligand (RANKL) and decreased osteoprotegerin (OPG), which aggravates bone loss (Huang et al., 2004; Jilka et al., 2010). In addition, the T cells-mediated bone resorption can be triggered by cPTH, and enhanced production of TNF is detected when targeting T cells with PTH (Gao et al., 2008; Li et al., 2015). On the contrary, intermittent PTH (iPTH) treatment elevates bone mass by activating the expansion and differentiation of osteoblasts (Nishida et al., 1994; Terauchi et al., 2009; Kim et al., 2012; Wein and Kronenberg, 2018). PTH-related protein (PTHrP) is expressed by chondrocytes in the resting zone of growth plate (Abad et al., 2002). Owing to the structure homology, PTHrP and PTH bind to the shared G-protein coupled receptor, PTH1R, which is expressed on osteoblast (Datta and Abou-Samra, 2009). Consistently, intermittent treatment with PTHrP shows anabolic effects by stimulating the differentiation of subchondral bone marrow-derived mesenchymal stem cell (SMSC), initiating endochondral ossification, and promoting the early osteoblastic cells development (Huch et al., 2003; Datta et al., 2007; Zhang et al., 2022), while continuous PTHrP treatment leads to bone resorption (Teitelbaum, 2000).

The regulatory effects of certain probiotic strains on PTH level have been observed in rodent models and clinical trials (Narva et al., 2004; Takasugi et al., 2011; Jafarnejad et al., 2017; Fernández-Murga et al., 2020), indicating the critical role of gut microbiota in in PTH production. Recent studies have further revealed the essential roles of gut microbiota in bone metabolism regulated by PTH. The absence of bone anabolic effect of iPTH has been observed in axenic mice (Li et al., 2020). Further investigation has revealed that butyrate, one of the gut microbial metabolites, facilitates the expansion of circulating regulatory T cells (Tregs) through GPR43 signaling. Besides, butyrate can potentiate iPTH in inducing bone marrow Treg cells and stimulate the secretion of osteogenic Wnt10b from CD8^+^ T cells (Li et al., 2020). On the other hand, cPTH only elicits bone catabolic activity in the mice colonized with segmented filamentous bacteria (SFB), rather than axenic mice (Yu et al., 2020). SFB are spore-forming, Gram-positive commensal bacteria that colonize in the murine small intestine, and potentially provoke the intestinal T cell responses (Davis and Savage, 1974; Gaboriau-Routhiau et al., 2009). The presence of SFB promotes cPTH in inducing Th17 and TNF^+^ T cells development. The elevated level of TNF in bone marrow stimulates the migration of intestinal Th17 cells to the bone marrow, and Th17 cells further promotes the osteoclastogenesis by secreting IL-17A, RANKL, TNF, IL-1, and IL-6 (Yu et al., 2020). As for PTHrP, no evidence has so far implied its correlation with gut microbiota.

### 3.4. 5-hydroxytryptamine

Serotonin, also known as 5-hydroxytryptamine (5-HT), is a candidate hormone in bridging gut microbiota and bone homeostasis. Approximately 95% of body’s 5-HT is synthesized by enterochromaffin (EC) cells in the gastrointestinal tract under the control of tryptophan hydroxylase 1 (TPH1), while only a minor fraction is produced by brainstem spinal neurons (Gershon and Tack, 2007; Ducy and Karsenty, 2010). These 2 types of 5-HT play antagonistic functions in bone remodeling through different pathways. The peripheral 5-HT exerts negative effects on bone formation, for 5-HT not only suppresses the proliferation of osteoblast *via* Htr1b/PKA/CREB/cyclins signaling cascade, but also enhances the osteoclastic resorption (Yadav et al., 2008; Ducy and Karsenty, 2010; Chabbi-Achengli et al., 2012; Spohn and Mawe, 2017). The *Tph1^−/−^* mice exhibits ameliorated bone resorption as compared to the WT mice (Chabbi-Achengli et al., 2012). Consistently, TPH1 inhibition can rescue the bone loss in ovariectomized rodent models (Yadav et al., 2010; Inose et al., 2011). In addition, application of selective serotonin reuptake inhibitors (SSRIs), which are extensively used in treating psychiatric disorders, is associated with reduced BMD and bone formation marker (Feuer et al., 2015; Kumar et al., 2018; Wu et al., 2018). Thus, the inhibition of gut-derived 5-HT is considered as a potential treatment for the bone-loss disorder.

Emerging evidences indicate that the process of ECs producing 5-HT is microbiota-dependent. Down-regulated gene expression of TPH1 with deficient peripheral 5-HT level have been observed in GF mice, accompanied by improved bone mass (Wikoff et al., 2009; Yano et al., 2015). The reconstitution of gut microbiota in GF mice effectively restores the peripheral 5-HT concentration (Yano et al., 2015; Yan et al., 2018). Spore-forming bacteria (Sp) plays pivotal role in regulating EC cells’ function and 5-HT biosynthesis (Yano et al., 2015; Wu et al., 2018). In addition, microbes including *Candida* spp., *Streptococcus* spp., *Escherichia* spp., and *Enterococcus* spp., also facilitate 5-HT production (Cryan and Dinan, 2012). In addition, excessive ethanol consumption is recognized as an important risk factor for osteopenia (Luo et al., 2017; Wu et al., 2018), as gut microbiota dysbiosis caused by chronic ethanol abuse leads to elevated 5-HT concentration and aggravated bone resorption (Liu et al., 2022). In contrast to peripheral 5-HT, the brain derived central 5-HT acts as a neurotransmitter which promotes bone formation *via* binding to Htr2c receptors on ventromedial hypothalamic neurons (Yadav et al., 2009). Although no direct link between gut microbiota and central 5-HT has been demonstrated, propionic acid derived from gut bacteria can significantly reduce the brain 5-HT in Western Albino rats (Al-Ghamdi et al., 2014).

### 3.5. Gastrointestinal hormones

The gastrointestinal (GI) tract is an important site of endocrine production, and signaling molecules released from enteroendocrine cells (EECs) act on regulating digestive effect and energy metabolism. Since the clinical symptom of low BMD occasionally occurs after bariatric surgery or during gastrointestinal diseases (Lehto-Axtelius et al., 2002; Brzozowska et al., 2013), the involvement of gastrointestinal hormones in bone homeostasis has drawn increasing attention over the years. In addition, accumulating evidences have demonstrated that functions of EECs are under the regulation of gut microbiota, and the microbiota- hormone interaction is of great importance in the gut-bone axis (Furness et al., 2013; Viswanathan, 2013; Ye et al., 2021).

#### 3.5.1. Ghrelin

Ghrelin is a 28 amino acid peptide mainly synthesized in the stomach. Ghrelin was initially identified as the endogenous ligand of GH secretagogue receptor (GHSR), acting as an appetite stimulant and energy regulator (Kojima et al., 1999; Perelló and Zigman, 2012; Huang et al., 2017). In addition to its bone anabolic effect *via* stimulating GH secretion (Kojima et al., 1999), the GH-independent effects of ghrelin on skeletal metabolism have also been identified. By binding to the GHSR, ghrelin directly stimulates the proliferation and differentiation of osteoblasts (Fukushima et al., 2005; Kim et al., 2005; Maccarinelli et al., 2005), and the mitogen-activated protein kinase (MAPK)/phosphoinositide 3-kinase (PI3K) pathway is involved in this process (Delhanty et al., 2006). However, a negative association between serum ghrelin level and bone mass or bone synthesis marker PINP has been observed in either female suffered from anorexia nervosa or obesity patients who have weight loss, and this is probably attributed to the elevated adrenocorticotropic hormone (ACTH) level induced by ghrelin (Arvat et al., 2001; Misra et al., 2005; Yu et al., 2022).

The interaction between gut microbiota and ghrelin seems controversial. Patients with polycystic ovary syndrome (PCOS) exhibit gut microbial dysbiosis accompanied with decreased serum ghrelin (Liu et al., 2017). A case–control study has revealed that the ghrelin level is negatively correlated with fecal *Bifidobacterium*, *Lactobacillus* and *Blautia coccoides-Eubacterium rectale* group, but positively correlated with *Bacteroides* and *Prevotella* (Queipo-Ortuño et al., 2013). The absence of gut microbiota perturbs the secretion of ghrelin (Perry et al., 2016; Weger et al., 2019), while increased acetate production induced by colonization of gut microbiota to GF mice activates the parasympathetic nervous system and enhances ghrelin secretion (Perry et al., 2016). However, a recent study has shown the attenuating effects of SCFAs on ghrelin-mediated signaling through the GHSR-1a, suggesting a negative effect of gut microbiota in ghrelin-induced activity (Torres-Fuentes et al., 2019).

#### 3.5.2. Incretins

Incretins are a group of insulin-tropic peptides synthesized in small intestine, responding to nutrient intake and stimulating insulin secretion (Baggio and Drucker, 2007; Nauck and Meier, 2018). The most well-known incretins are glucose-dependent insulinotropic polypeptide (GIP) and glucagon-like peptide-1 (GLP-1), which are secreted by K-cells and L-cells respectively, and serve as anabolic signals to the skeletal system. GIP receptors (GIPRs) are widely expressed on osteoblastic lineage and osteoclasts (Bollag et al., 2000), and the activation of GIPRs leads to the release of bone formation markers, inhibition of osteoblasts apoptosis, and suppression of osteoclast functions (Zhong et al., 2007; Berlier et al., 2015; Mabilleau et al., 2016; Stensen et al., 2020). The beneficial effects of GIP on bone have also been observed *in vivo*, as GIP administration effectively prevents bone resorption (Bollag et al., 2001; Nissen et al., 2014), while GIPR knockout mice exhibits defective bone property and suppressed bone formation (Xie et al., 2005; Gaudin-Audrain et al., 2013; Mieczkowska et al., 2013). GLP-1 was previously believed to attenuate bone resorption by stimulating calcitonin production (Yamada et al., 2008). However, GLP-1 receptors (GLP-1Rs) are widely expressed on osteoblastic cell lines, bone marrow-derived macrophage (BMM), and preosteoclast, and GLP-1 can not only enhance the viability of osteoblast (Pacheco-Pantoja et al., 2011), but also inhibit osteoclastogenesis through the NF-κB/MAPK-nuclear factor of activated T cells (NFATc1) pathway (Li et al., 2020). Additionally, administration of GLP-1 to adipose-derived stem cells (ADSCs) stimulates the osteoblast differentiation (Lee et al., 2015), and upregulated expression of GLP-1R has been detected during osteogenesis (Jeon et al., 2014). The GLP-1 receptor knockout mice exhibit reduced bone quality but increased bone resorption (Yamada et al., 2008; Mabilleau et al., 2013), while GLP-1 treatment improves bone structure in streptozotocin-induced diabetic Wistar rats and hyperlipidemic rats (Nuche-Berenguer et al., 2009, 2011).

Studies have shown that administration of probiotics or prebiotics elevates the serum level of GLP-1 (Shirouchi et al., 2016; Kim et al., 2018; Wang et al., 2020). SCFAs can trigger the secretion of GLP-1 by activating SCFA receptors FFAR2 (GPR43) and FFAR3 (GPR41) in L cells (Tolhurst et al., 2012). Interestingly, elevated level of GLP-1 has also been observed in microbiota-deleted mice as compared to the conventionally raised mice (Wichmann et al., 2013; Zarrinpar et al., 2018; Heiss et al., 2021), and this increment is possibly attributed to the suppressed intestinal transit and altered colonocyte glucose utilization caused by SCFAs deficiency (Donohoe et al., 2011; Zarrinpar et al., 2018).

#### 3.5.3. Peptide YY

Belonging to the neuropeptide Y peptide family, peptide YY (PYY) is predominantly secreted from L-cells, and plays pivotal roles in regulating appetite and energy homeostasis (Batterham et al., 2002; Batterham and Bloom, 2003). PYY is regarded as a negative regulator in bone metabolism. A negative correlation between circulating PYY level and BMD has been observed in patients of anorexia nervosa, patients receiving bariatric surgery and premenopausal women (Utz et al., 2008; Scheid et al., 2011; Yu et al., 2016; Kim et al., 2020). PYY binds to Y-receptors, particularly Y1R and Y2R, with high affinity, and Y1R^−/−^ mice exhibit enhanced osteoblastic activity with increased mineral apposition (Lee et al., 2011). However, the deletion of PYY in mice leads to controversial skeletal phenotypes. Replacement of the *Pyy* coding region with a *lacZ* reporter gene results in a decreased BMD (Wortley et al., 2007), while either retaining *Cre* recombinase gene and *EGFP* reporter gene or deletion of the entire PYY coding region promotes the bone formation (Wong et al., 2012; Leitch et al., 2019). These divergent phenotypes may be attributed to different mice age and gene manipulation approaches. The production of PYY is under the regulation of gut microbiota. The ceftazidime (an anti-Gram-negative bacteria antibiotic) treatment significantly promotes PYY secretion in high-fat diet feed mice (Rajpal et al., 2015). By activating Toll-like receptors (TLRs) in L-cells, the microbiota-derived butyrate strongly promotes the *Pyy* expression (Larraufie et al., 2017). Moreover, the bile acid G protein-coupled receptor, Takeda G protein receptor 5 (TGR5), stimulates the release of PYY, while H_2_S, produced by sulfate-reducing commensal bacteria in colon, inhibits the TGR5-dependent PYY release involving PLC-ε/Ca^2+^ pathway (Bala et al., 2014).

### 3.6. Adipocytokines

Accumulating evidences have revealed the intimate correlation between adipocytes-derived hormones and skeletal homeostasis. Leptin is considered as a crucial signal in regulating food intake and energy expenditure. By binding to the receptors on bone marrow mesenchymal stem cells (BMSCs) with high affinity, leptin promotes the proliferation and the differentiation of BMSC into the osteoblastic lineage (Thomas et al., 1999; Astudillo et al., 2008), and further stimulates the osteoblast development and bone mineralization (Reseland et al., 2001). In addition to the direct effect on bone cells, leptin exerts beneficial effects on skeletal health by facilitating GH secretion (Carro et al., 1997; Tannenbaum et al., 1998). Adiponectin is an adipocytokine that sensitizes the body to insulin (Kadowaki et al., 2006). Adiponectin has been shown to stimulate the differentiation of osteoblasts *via* MAKP-cyclooxygenase-2 (COX2) signaling (Luo et al., 2005; Lee et al., 2009). Moreover, leptin and adiponectin also show inhibitory effects on osteoclastogenesis (Holloway et al., 2002; Tu et al., 2011). Leptin level may also be regulated by gut microbiota, as positive correlations of leptin with *Bifidobacterium* and *Lactobacillus*, and negative correlations with *Clostridium*, *Bacteroides* and *Prevotella* have been demonstrated in rats under different nutritional status and physical activity (Queipo-Ortuño et al., 2013).

Taken together, although no direct causal effects between gut microbiota and endocrine system in regulating skeletal homeostasis have been well demonstrated, current evidence has shown strong intercorrelations between gut microbiota and the production and activity of hormones related to bone health. Further delineation of the underlying mechanisms by which gut microbiota interplays with endocrine system may provide novel target for the better management of skeletal disorders.

## 4. How does gut microbiota regulate the enteroendocrine-osseous axis

### 4.1. SCFAs

**Figure 1:**
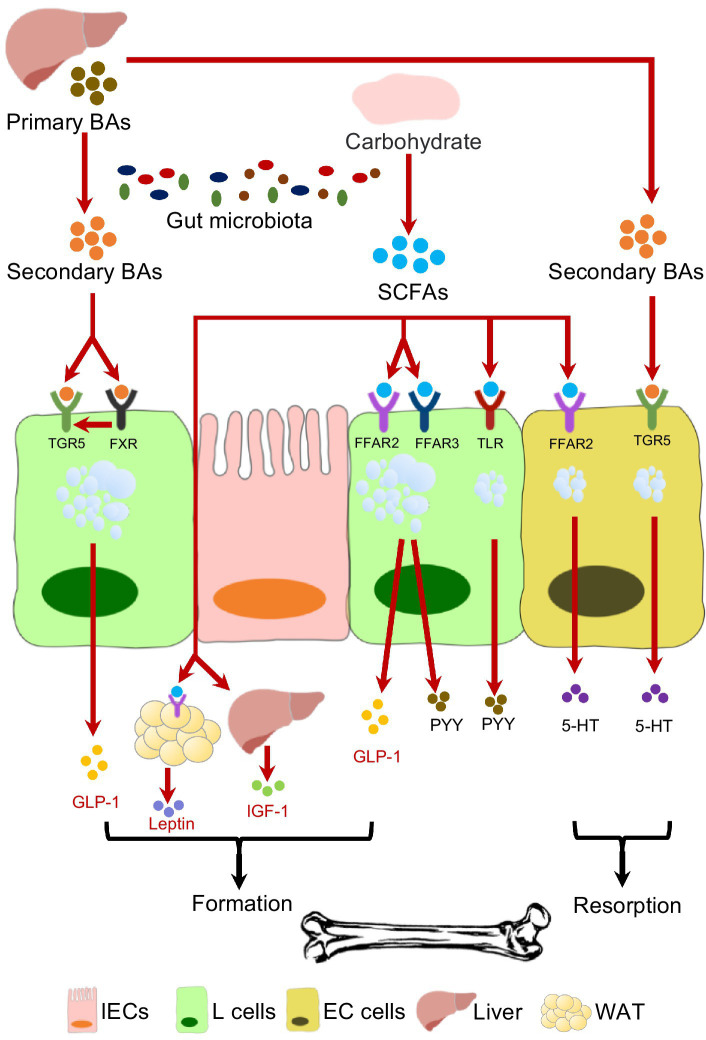
The effects of gut microbiota on endocrine-osseous axis mediated by SCFAs and BAs.

One of the important factors by which gut microbiota interplays with endocrine system is the production of SCFAs (Figure 1). Acetate, butyrate and propionate are the most common SCFAs generated from microbial fermentation of non-digestible polysaccharides. The involvement of SCFAs in skeletal homeostasis has been well reported in recent years (Zaiss et al., 2019; Wallimann et al., 2021). It has been demonstrated that SCFAs suppress the osteoclasts differentiation *via* inhibiting histone deacetylases (HDACs) and activating FFARs (Rahman et al., 2003; Kim et al., 2018; Yan et al., 2018; Montalvany-Antonucci et al., 2019). Additionally, SCFAs stimulate bone formation by increasing the production of bone sialoprotein (BSP) and osteopontin (OPN) from osteoblasts (Katono et al., 2008). Despite the direct effects on bone cells, SCFAs facilitate Tregs cells development (Furusawa et al., 2013; Smith et al., 2013; Dalile et al., 2019), and in the meantime inhibit osteoclastogenic Th17 cells (Asarat et al., 2016; Luu et al., 2019), maintaining the osteo-immune homeostasis. The administration of probiotics or berberine has been reported to effectively elevate the gut SCFAs level, leading to alleviated bone resorption (Jia et al., 2019, 2021).

SCFAs are capable of selectively promoting L-cell proliferation (Kaji et al., 2011; Petersen et al., 2014), which facilitate the enteroendocrine hormone production. SCFA receptors, especially FFAR2 and FFAR3, are expressed on L-cells that are involved in enteroendocrine hormones secretion (Karaki et al., 2006; Tazoe et al., 2009). SCFAs enhance the production of GLP-1and PYY by activating L-cells through FFARs (Lin et al., 2012; Brooks et al., 2017), and mice lacking of FFAR2 or FFAR3 secret reduced GLP-1 (Samuel et al., 2008; Tolhurst et al., 2012). The impacts of SCFAs on 5-HT secretion seem to be bone-detrimental. SCFAs can stimulate gut 5-HT release in rodent model by activating FFAR2 (Fukumoto et al., 2003; Akiba et al., 2015), and SCFAs can also promote the gene expression of *Tph1* (Yano et al., 2015). Moreover, propionate is able to induce the brain toxication and deplete central 5-HT production (Al-Ghamdi et al., 2014). In addition to the well-recognized roles of gut microbiota in producing SCFAs, the expression of FFAR gene can also be regulated by gut microbiota (Han et al., 2021; Zhang et al., 2021).

The production of adipocytes-derived leptin is also stimulated by SCFAs, and Galpha(i) signaling is involved in this process (Zaibi et al., 2010). As for IGF-1, a concomitant decrease of gut SCFAs level and systemic IGF-1 level have been reported in antibiotic-treated mice, while SCFAs supplementation restores the IGF-1 secretion (Yan et al., 2016). However, no evidence has yet confirmed the involvement of FFARs in SCFAs-mediated IGF-1 production. However, the production of some hormones is suppressed by SCFAs. A decreased ghrelin level and attenuated ghrelin receptor signaling were observed with the application of SCFAs (Fukumori et al., 2011; Torres-Fuentes et al., 2019). The inhibition of GIP secretion was also conducted by SCFA binding FFAR3 (Lee et al., 2018), even though SCFAs facilitated the K-cells expansion.

### 4.2. Bile acids

Bile Acids (BAs) are another major contributor to the intercorrelations between gut microbiota and endocrine system in maintaining skeletal homeostasis (Figure 1). The primary bile acids (BAs), including cholic acid (CA) and chenodeoxycholic acid (CDCA), are cholesterol-derived metabolites synthesized in the liver, and then enter the duodenum through bile duct. Microbial degradation further coverts primary BAs to secondary BAs, such as lithocholic acid (LCA) and deoxycholic acid (DCA) (Russell, 2003). The systemic BA homeostasis is under the feedback control of farnesoid X receptor (FXR)-fibroblast growth factor 15 (FGF15) axis, which requires the presence of gut microbiota (Sinal et al., 2000; Chiang, 2013; Wang and Yao, 2021). Anaerobic bacteria such as *Bacteroides*, *Eubacterium*, and *Clostridium* are involved in the BAs metabolism (Narushima et al., 2006; Nicholson et al., 2012; Long et al., 2017; Wang and Yao, 2021). A positive correlation between serum level of total BAs and BMD has been reported in the postmenopausal women (Zhao et al., 2020), and FXR^−/−^ mice exhibits reduced BMD (Cho et al., 2013), indicating the critical role of BAs in bone remodeling. BAs not only facilitate osteoblast formation by up-regulating the Runx2 expression and enhancing extracellular signal-regulated kinase (ERK) (Cho et al., 2013), but also promote the osteogenic differentiation of bone marrow-derived mesenchymal stem cells (BMMSCs) by regulating Integrin 5 (ITGA5) (Cha et al., 2016). Despite the direct effects on bone cells, FXR and G protein-coupled bile acid receptor-1 (Gpbar-1, also known as TGR5) are involved in BAs-stimulated GLP-1 and PYY secretion (Katsuma et al., 2005; Bala et al., 2014; Pathak et al., 2018). Lithocholic acid-producing bacteria including *Acetatifactor* and *Bacteroideswere* have been found to modulate FXR/TGR5/GLP-1 signaling (Pathak et al., 2018). However, negative effects of BAs on bone metabolism have been also reported. DCA can upregulate *tph1* expression by activating TGR5, and thus promote gut 5-HT release, which may result in bone resorption (Alemi et al., 2013; Wang and Yao, 2021). FGF15 is also identified as a gut hormone, and the FGF15 knockout mice exhibits osteopenia phenotype, suggesting its positive effect in bone remodeling (Bozadjieva-Kramer et al., 2021). Application of antibiotics minocycline can lead to impaired skeletal maturation in young mice, likely due to excessive serum conjugated BAs resulted from gut microbiota dysbiosis and disrupted FXR-FGF15 axis (Carson et al., 2022).

### 4.3. Other mediators

The LPS derived from Gram-negative bacteria is known to be a Toll-like receptor (TLR) 4 ligand. By activating TLR4, LPS can stimulate 5-HT production from EC cells (Kidd et al., 2009). In addition to SCFAs, other bacterial metabolites including α-tocopherol, cholate, deoxycholate, p-aminobenzoate (PABA) and tyramine are also capable of stimulating gut-derived 5-HT synthesis (Yano et al., 2015). Furthermore, a recent study reported that extracellular RNAs may serve as an activator of ion channels. By sensing single-stranded RNA (ssRNA) from gut microbiota, Piezo1, a mechanosensitive Ca^2+^ channel in ECs, can be activated and subsequently triggers the transcription of *Thp1* and production of 5-HT (Sugisawa et al., 2020).

Therefore, current evidence has suggested that bacterial metabolites, particularly SCFAs, are the major molecular basis by which gut microbiota regulates the enteroendocrine-osseous axis. In addition, bile acid homeostasis, which is closely associated with gut microbiota, also involved in the secretion of bone-related hormones such as 5-HT and GLP-1. Modulation of gut microbiota to promote SCFAs production and maintain a bile acid homeostasis is promising to a joint management of skeletal and endocrine disorders.

## 5. Potential therapeutic interventions on enteroendocrine-osseous axis

### 5.1. Probiotics

Probiotics are live microorganisms that benefit to human health when consumed in adequate amounts (Pineiro and Stanton, 2007). Mainly by elevating luminal SCFA levels, probiotics administration is capable of reducing intestinal permeability, alleviating inflammatory response and promoting osteoblast formation, thus exerting protective effects against bone loss (Ibáñez et al., 2019; Zaiss et al., 2019; Jia et al., 2021). Relevant studies on the anabolic effect of probiotics *via* regulating endocrine system are listed Table 1. GH and IGFs are the most commonly affected hormones by probiotics. *Lactobacillus plantarum* has been shown to sustain the growth of *Drosophila* and mice under the nutrition scarcity by promoting the production of dILPs or IGF-1 (Storelli et al., 2011; Schwarzer et al., 2016). Likewise, the beneficial impacts of probiotics on bone traits have been demonstrated in zebrafish and broiler chicken, accompanied with enhanced IGF and GH level (Avella et al., 2012; Abdelqader et al., 2020). Probiotics can also regulate PTH production. It has been reported that *Bifidobacterium pseudocatenulatum* can effectively reduce serum PTH level and meanwhile improve the trabecular architecture in mice fed with high fat diet (Fernández-Murga et al., 2020). In addition, *Bacillus subtilis* can reduce bone resorption in broilers under thermoneutral conditions, which is mediated by increased central 5-HT level (Yan et al., 2018). The regulatory effects of probiotics on GLP-1, leptin and adiponectin have also been reported, however, the relevant bone phenotypes have not been reported yet (Bagarolli et al., 2017; López-Moreno et al., 2020; Wang et al., 2020).

**Table 1:** The regulatory effect of probiotics on enteroendocrine-osseous axis.

Probiotics strains	Animals	Target hormones	Bone phenotypes	References
*Lactobacillus plantarum* WJL	*Drosophila*	dILPs ↑	Growth rate ↑	Storelli et al. (2011)
*Lactobacillus plantarum* WJL, NIZO2877	BALB/c Mice	GH, IGF-1and IGFBP-3 ↑	Body length and femur length ↑	Schwarzer et al. (2016)
*Lactobacillus rhamnosus* IMC 501	Zebrafish	IGF-1and IGF-2 ↑	Backbone calcification ↑	Avella et al. (2012)
*Bacillus subtilis*	Broiler chicken	GH, IGF-1 ↑	Tibia weight, length, density and ash content ↑	Abdelqader et al. (2020)
*Bacillus subtilis*	Broiler chicken	Central 5-HT ↑	BMD of tibia and femur ↑	Yan et al. (2018)
*Bifidobacterium pseudocatenulatum* CECT 7765	C57BL-6 mice	PTH ↓	Trabecular architecture (volumetric fraction, trabecular number and trabecular pattern factor) ↑	Fernández-Murga et al. (2020)

### 5.2. Prebiotics

Prebiotics are defined as selectively fermented ingredients, which lead to specific gut microbiota alteration and confer benefits upon host health (Sheveleva, 1999). Non-digestible oligosaccharides (NDOs), such as galactooligtosaccharides (GOS), fructooligosaccharidesc (FOS), oligofructose, and inulin are regarded as the most beneficial prebiotics to bone health. Application of FOS, GOS and inulin not only promotes growth (Scholz-Ahrens et al., 2007; Edwards et al., 2020), but also effectively prevent the bone loss induced by ovarian hormone deficiency (Mathey et al., 2004; Zafar et al., 2004; Hooshmand et al., 2010; Seijo et al., 2019). Prebiotics also exert favorable impacts on bone health by regulating microbial ecology, promoting SCFAs production, and facilitating nutrition absorption (Scholz-Ahrens et al., 2007; Edwards et al., 2020). In addition, consumption of prebiotics elevates the level of hormones including IGF-1, IGF-2, and GH (Fordjour et al., 2010; Cluny et al., 2015; Kareem et al., 2016; Soumeh et al., 2019), which shows beneficiary effects on bone health. Although there still lacks direct evidence to support the interplay of prebiotics and hormones in maintaining bone health, a clinical study has shown that supplement of FOS-inulin to milk results in decreased serum level of PTH, accompanied with reduced bone resorption in postmenopausal women (Kruger et al., 2016).

### 5.3. Fecal microbiota transplantation

Considering the intimate correlation between gut microbiota and skeletal health, microbiota-targeted technique, such as fecal microbiota transplantation (FMT), has become a promising therapeutic strategy in the treatment of bone disorder. FMT refers to the transferring of feces from a healthy donor to the GI tract of a recipient patient, in order to treat a specific disease associated with gut microbiota disorder (Cammarota et al., 2017). Animal studies have demonstrated that FMT can alleviate bone lose by improving intestinal microenvironment and adjusting metabolic functions (Ma et al., 2021; Zhang et al., 2022). A recent study has shown that FMT can sustain IGF-1 production and attenuate bone destruction in sickle cell disease mice by increasing the level of SCFAs (Xiao et al., 2022). FMT can also effectively prevent the estrogen deprivation-induced bone loss, accompanied by elevated SCFAs and decreased pro-osteoclastogenic cytokines release (Zhang et al., 2022). However, cautions should be taken when performing FMT, as it may result in side effects including abdominal discomfort, constipation, diarrhea, etc. (Baxter and Colville, 2016; Wang et al., 2019). In addition, inappropriate donor recruiting or fecal material screening may cause the contamination of patient with pathogenic micro-organisms (Wang et al., 2019; Antushevich, 2020).

### 5.4. Bacteriophage therapy

Bacteriophages (phages) are viruses that infect bacteria, and can kill the host by its replication within infected bacterium and lysis (Wommack and Colwell, 2000). Since antibiotic resistance has become a severe problem in orthopedics, typically implant-associated infection (Campoccia et al., 2006), phage therapy is now regarded as valuable adjunct. *In vitro* studies have demonstrated that the utilization of phage exhibits anti-biofilm effect by suppressing the bacterial growth and adhesion (Kaur et al., 2014; Morris et al., 2019), and phage is also effective in alleviating skeletal inflammation in animal studies (Yilmaz et al., 2013; Kaur et al., 2016). However, the phage therapy is generally given by local injection, further studies focused on the treatment of skeletal disorder mediated by gut microbiota are still needed.

In a word, the manipulation of gut microbiota has been proved as a practicable strategy in improving skeletal health, mainly by promoting the production of SCFAs. However, the involved mechanisms, such as target endocrine factors and biological pathways, still needs further investigation. In addition, since every individual owns unique gut microbiota, more precise medical treatment is wait to be developed.

## 6. Conclusion and prospects

In recent years, the regulatory effects of gut microbiota on bone metabolism have attracted increasing attention. Despite the well-recognized pathways that involve gut barrier and osteoimmunology, emerging evidence has indicated that gut microbiota can impact bone health *via* interplaying with the endocrine system of host. Gut microbiota profoundly influences the secretion of a variety of bone metabolism-related hormones including IGF-1, 5-HT, PTH, GLP-1 and leptin, which further regulate bone homeostasis. SCFAs and BAs are identified as important mediators of gut microbiota in regulating skeletal health *via* enteroendocrine-osseous axis. Supplementation of probiotics and prebiotics may serve as a promising adjuvant therapy in promoting bone health. However, more well-controlled clinical trials are still needed to translate these findings to the better management of skeletal disorders.

## Author contributions

XX designed and structured the manuscript. YT performed the literature search and wrote the draft of the manuscript. XK and LZ critically revised the manuscript. XX revised and edited the final version of the manuscript. All authors have read and agreed to the published version of the manuscript.

## Funding

This study was supported by the grant from Science and Technology Department of Sichuan Province (2021YFQ0064), and the grant from Health Commission of Sichuan Province (21PJ058).

## Conflict of interest

The authors declare that the research was conducted in the absence of any commercial or financial relationships that could be construed as a potential conflict of interest.

## Publisher’s note

All claims expressed in this article are solely those of the authors and do not necessarily represent those of their affiliated organizations, or those of the publisher, the editors and the reviewers. Any product that may be evaluated in this article, or claim that may be made by its manufacturer, is not guaranteed or endorsed by the publisher.
